# Abyssal deposit feeders are secondary consumers of detritus and rely on nutrition derived from microbial communities in their guts

**DOI:** 10.1038/s41598-021-91927-4

**Published:** 2021-06-15

**Authors:** Sonia Romero-Romero, Lee C. Miller, Jesse A. Black, Brian N. Popp, Jeffrey C. Drazen

**Affiliations:** 1 Department of Oceanography, University of Hawaii at Manoa, 1000 Pope Road, Honolulu, HI 96822 USA; 2 Department of Earth Sciences, University of Hawaii at Manoa, 1680 East West Road, Honolulu, HI 96822 USA

**Keywords:** Stable isotope analysis, Ecology, Ocean sciences

## Abstract

Trophic ecology of detrital-based food webs is still poorly understood. Abyssal plains depend entirely on detritus and are among the most understudied ecosystems, with deposit feeders dominating megafaunal communities. We used compound-specific stable isotope ratios of amino acids (CSIA-AA) to estimate the trophic position of three abundant species of deposit feeders collected from the abyssal plain of the Northeast Pacific (Station M; ~ 4000 m depth), and compared it to the trophic position of their gut contents and the surrounding sediments. Our results suggest that detritus forms the base of the food web and gut contents of deposit feeders have a trophic position consistent with primary consumers and are largely composed of a living biomass of heterotrophic prokaryotes. Subsequently, deposit feeders are a trophic level above their gut contents making them secondary consumers of detritus on the abyssal plain. Based on δ^13^C values of essential amino acids, we found that gut contents of deposit feeders are distinct from the surrounding surface detritus and form a unique food source, which was assimilated by the deposit feeders primarily in periods of low food supply. Overall, our results show that the guts of deposit feeders constitute hotspots of organic matter on the abyssal plain that occupy one trophic level above detritus, increasing the food-chain length in this detritus-based ecosystem.

## Introduction

Detritus is the main standing stock of organic matter for most ecosystems, both aquatic and terrestrial^1^, and its importance in ecosystem functioning has long been noted^2^. Since detritus is the most abundant source of carbon at the base of food webs^3^, decomposers are the dominant feeding guild on Earth^4^. Most decomposers are prokaryotes and small invertebrate detritivores^5^. Thus, detritus-based food webs have highly complex trophic-dynamics and, contrary to primary producer-based food webs, are difficult to compartmentalize into trophic levels. As a consequence, trophic ecology has largely neglected the inclusion of detritus and its pathways into higher trophic levels^6,7^, and instead they often appear illustrated as a black box in the flow of carbon or energy through food webs.

Most deep-sea ecosystems, where there is no in situ primary production (except for chemoautotrophy at e.g. hydrothermal vents and cold seeps), entirely depend on detritus arriving from surface waters^8–10^. Detritus is mainly consumed as it sinks through the water column before reaching the deep seafloor (on average only < 5% of surface production reaches abyssal depths^9,11^), so these ecosystems are largely limited by food, which often arrives with marked seasonality^12^. The deep ocean is the largest ecosystem on Earth, and within it the abyssal seafloor occurring between 3000 and 6000 m depth, covers 54% of the Earth’s surface^13^. Abyssal plains can host high faunal diversity and given their vast size are a major reservoir of biodiversity^9^, which can exert a significant influence on the carbon cycle. Thus, understanding the energy flow within the deep-sea detrital food web and the trophic ecology of abyssal species is of global importance.

Deposit feeders are adapted to live in such food-poor environments and unlike many ecosystems, on the abyssal plains they can be very large, dominating megafaunal communities^10,14,15^. Deposit feeders play a key role in abyssal ecosystems since they rapidly consume detritus reaching the seafloor and are major sediment bioturbators^16–18^. They employ an array of feeding and digestive strategies that give them a competitive advantage to cope with food scarcity^19,20^. Some species of mobile deposit feeders selectively feed on freshly deposited patches of organic matter^20–22^. In other species the gut anatomy allows for efficient processing of ingested food by gut microbes^23^. In this sense, the guts of some deep-sea holothurians have been found to hold high abundance of prokaryotes relative to the surrounding sediments^23–25^. These microbial communities seem to increase the efficiency of deposit feeders to exploit the limiting resources, however, their functional role and whether they are selected from the environment or have a symbiotic origin remain unknown. It has been suggested that these microbial communities act as “commensal” flora to the deposit feeder^25^, possibly transforming refractory organic matter into compounds easier to digest^26^, or they might be used as a nutritional source for the deposit feeder^25,27^. Overall, gut microbial communities might be a crucial yet overlooked component of abyssal food webs.

In recent years, compound-specific stable isotope analysis of amino acids (CSIA-AA) has proven useful in estimating trophic position of metazoans more accurately than bulk isotope analysis^28,29^. The calculation of trophic position using CSIA-AA is based on the differential enrichment in ^15^N of amino acids (AAs), which can be grouped into “source” and “trophic” AAs^30^. “Trophic” AAs, like glutamic acid, increase their δ^15^N values with trophic level, whereas “source” AAs, like phenylalanine, reflect the δ^15^N values at the base of the food web, changing little with trophic transfer^31,32^. The difference in δ^15^N values between glutamic acid and phenylalanine of an organism is used to estimate its trophic position. Thus, by applying CSIA-AA, information about trophic position and the δ^15^N values at the base of the food web can be obtained from a single sample. This technique has also been used to prove that in laboratory-cultured heterotrophic bacteria, metabolic reactions can result in changes of δ^15^N values in AAs similar to metazoans^33,34^. Moreover, δ^15^N values of “source” AAs and δ^13^C values of essential AAs provide information on dietary sources in consumers. CSIA-AA can therefore be used to estimate trophic position of decomposers and can help to disentangle trophic dynamics of detritus-based food webs.

In this study we investigated the trophic ecology of abyssal deposit feeders and the role of microbial assemblages present in their guts. We suggest that gut microbes play an important role in supplying nutrition to deposit feeders. For that, we estimated the trophic position of three species of deposit feeders collected from the abyssal plain of the Northeast Pacific (Station M; ~ 4000 m depth), their gut contents and the surrounding sediments using the nitrogen isotopic composition of amino acids. Furthermore, we investigated the role of gut microbes in deposit feeder nutrition using the carbon isotopic composition of essential amino acids. Our findings contribute to the understanding of the trophic dynamics of this deep-sea ecosystem and reveal that microbes present in the guts of deposit feeders might support a detritus-based food web that is longer than expected.

## Results

### Trophic position

**Figure 1:**
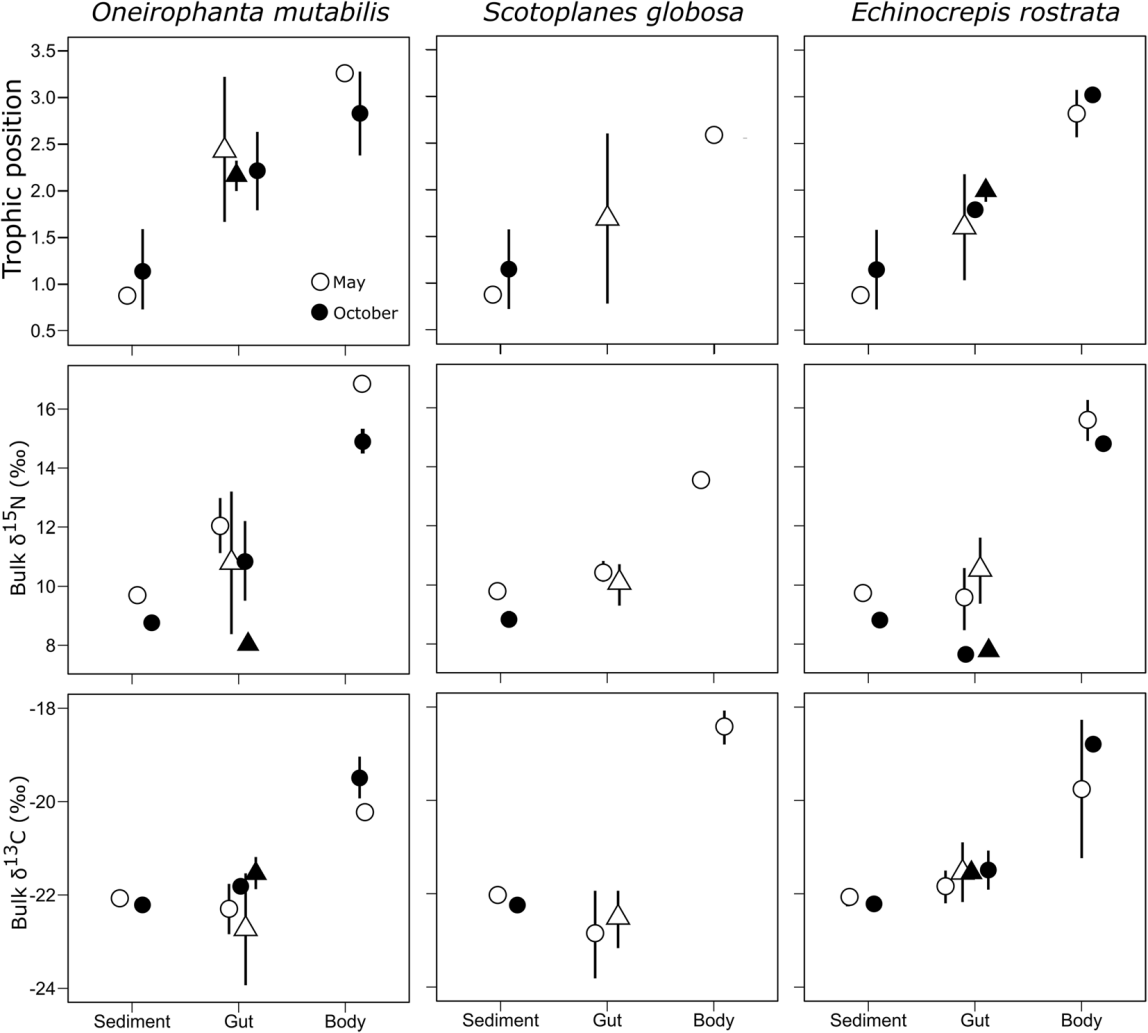
Trophic position, bulk δ^15^N and δ^13^C values for *O.* *mutabilis*, *S.* *globosa* and *E.* *rostrata*, their gut contents and the surrounding sediments, collected in the period of low food supply (i.e. May; open symbols) or in the period of high food supply (i.e. October; closed symbols). Within gut contents samples foregut (circles) or hindgut (triangles) are shown separately. Note that the same data from sediments are represented in all columns for comparison.

The average trophic position (TP), calculated from the AAs Glx and Phe, was 1.0 ± 0.2 (mean ± SD) for sediments. This confirms that a β value of 3.4‰ used in the calculation of trophic position was appropriate (see “Methods”). TP of deposit feeders (DF) was 2.9 ± 0.6 (Supplementary material, Table S1). Specifically, TP was 1.7 ± 0.1, 2.0 ± 0.2, and 2.4 ± 0.0 higher than that of surface sediments for *S.* *globosa*, *E.* *rostrata*, and *O.* *mutabilis,* respectively. Thus, deposit feeders were secondary consumers of detritus present in the surrounding sediments. Both foregut and hindgut contents were approximately one trophic level higher than sediments (ΔTP [≡ TP_gut content_ – TP_sediment_]; ΔTP_Hindgut_ = 0.8 ± 0.6 for *E.* *rostrata,* 0.8 ± 0.9 for *S.* *globosa* and 1.3 ± 0.6 for *O.* *mutabilis;* ΔTP_Foregut_ = 0.65 for *E.* *rostrata,* and 1.1 ± 0.4 for *O.* *mutabilis;* Fig. 1). Thus, trophic position of foregut contents were not significantly different from those of hindguts (ANOVA, F = 0.057, P > 0.05).

Bulk δ^15^N values of body tissue were also higher than those of sediments for the three species (Δδ^15^N [≡ δ^15^N_deposit feeder_—δ^15^N_sediment_] = 3.8‰ for *S.* *globosa,* 5.9‰ for *E.* *rostrata,* and 7.2‰ *O.* *mutabilis*); however, bulk δ^15^N values of gut contents were not different from those of sediments (ANOVA, F = 1.15, P > 0.05). Similarly to δ^15^N values, bulk δ^13^C values were comparable between sediments and gut contents but were higher for body tissue (Δδ^13^C [≡ δ^13^C _deposit feeder_—δ^13^C _sediment_] = 3.6‰ for *S.* *globosa,* 2.8‰ for *E.* *rostrata,* and 2.2‰ *O.* *mutabilis*). Trophic position was not significantly different between seasons for any species (Tukey test, P > 0.05) although *O.* *mutabilis* had a mean TP of 3.3 ± 0.0 and 2.8 ± 0.4 in periods of low and high food supply, respectively. However, bulk δ^15^N values of gut contents of all individuals collected were lower in the more productive period for both *O.* *mutabilis* (ANOVA, F = 8.19, P < 0.05, *n* = 8) and *E.* *rostrata* (ANOVA, F = 11.21, P < 0.05, *n* = 8).

### Elemental composition of sediment and gut contents

The contribution of total nitrogen (TN) to surface sediments was 0.22 weight (wt.) % for both periods. The contribution of TN in the hindgut contents of DFs was twice as high, ranging from 0.32 wt.% to 0.51 wt.%. In the foregut of *O.* *mutabilis* it was three and four times higher in periods of low and high food supply, respectively (0.60 wt.% and 0.85 wt.%; Table 1). Within TN, which accounts for both inorganic and organic N, the contribution of N from AAs was between 18 and 35 times higher inside the guts than in the sediments (Table 1).

**Table 1:** Total organic carbon (TOC), total nitrogen (TN) and contribution of N (% dry weight) from AAs to TN in surface sediments (0–5 mm) and gut contents (F: foregut; H: hindgut) of deposit feeders (mean ± SD) collected in periods of low food supply (i.e. May) and high food supply (i.e. October). The sample size was two for each mean value.

		%TOC		% TN		% AA/TN	
		Low food supply	High food supply	Low food supply	High food supply	Low food supply	High food supply
Sediment		1.4 ± 0.05	1.6 ± 0.02	0.22 ± 0.01	0.22 ± 0.02	0.28 ± 0.17	0.31 ± 0.09
*O.* *mutabilis*	F	3.7 ± 1.0	5.8 ± 0.07	0.60 ± 0.23	0.83 ± 0.11		5.76 ± 2.05
	H	3.2 ± 1.4	3.5 ± 0.8	0.47 ± 0.27	0.46 ± 0.14	10.83 ± 3.39	5.48 ± 0.55
*S.* *globosa*	F	3.0 ± 1.0		0.43 ± 0.15			
	H	2.1 ± 0.07		0.32 ± 0.03		3.88 ± 3.45	
*E.* *rostrata*	F	2.3 ± 0.5	3.4 ± 0.8	0.35 ± 0.01	0.42 ± 0.11		9.38 ± 3.47
	H	2.2 ± 0.4	2.9 ± 0.3	0.39 ± 0.09	0.51 ± 0.00	2.02 ± 0.34	10.97 ± 8.74

### δ^13^C values of essential AA and δ^15^N values of source AAs

**Figure 2:**
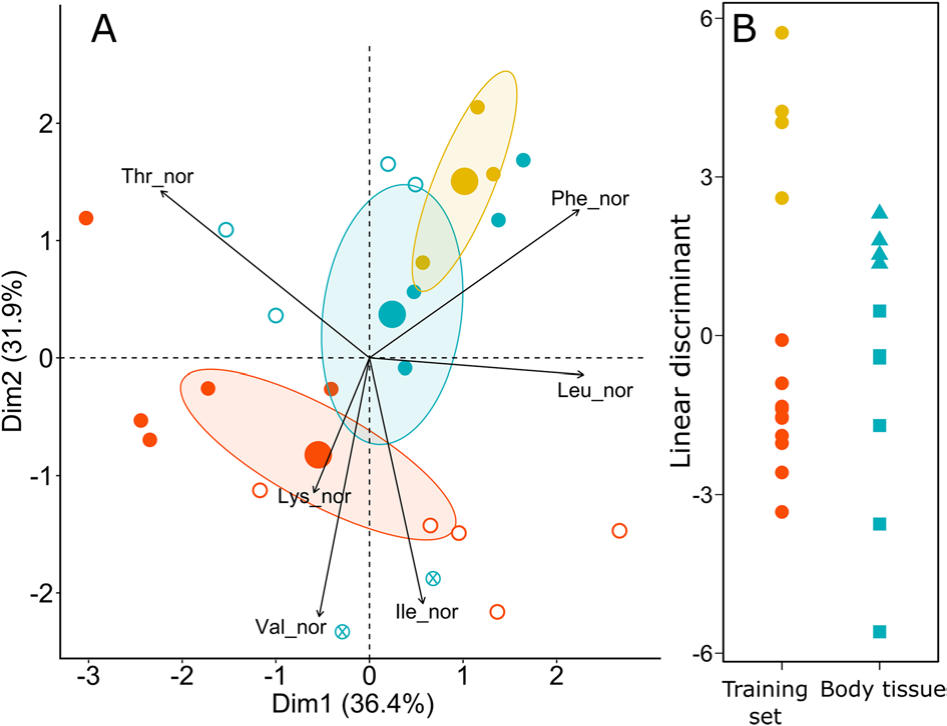
**(A)** Principal component analysis (PCA) using normalized δ^13^C values of essential amino acids (δ^13^C_EAA_) in surface sediments (yellow), gut contents (red) and body tissue (blue) of *O.* *mutabilis* (filled symbols), *E.* *rostrata* (open symbols), and body tissue of *S.* *globosa* (circled cross). Ellipses outline the 95% confidence region around the mean (large symbols) for each group. **(B)** Linear Discriminant Analysis (LDA) based on a training set built with δ^13^C_EAA_ values of surface sediments (yellow) and gut contents (red). Body tissue samples (blue) were collected in the period of low food supply (i.e. May; squares) and in the period of high food supply (i.e. October; triangles).

Results of PCA of normalized δ^13^C values of essential AAs (δ^13^C_EAA_) showed that PC1 and PC2 together explained 68.3% of the variation (Fig. 2). δ^13^C_EAA_ values were not available for gut contents of *S.* *globosa.* The normalized δ^13^C_EAA_ values differed significantly between sediments, gut contents and body tissue (PERMANOVA, P < 0.05). Phe and Val differed significantly between gut contents and body tissue, whereas Lys differed between sediments and gut contents (ANOVA, P < 0.05). After including gut contents and sediments as two food sources in the training set, the LDA grouped all DFs collected during low food supply with gut contents (probability > 99%), whereas during high food supply *E.* *rostrata* was grouped with sediments (prob. > 90%) and *O.* *mutabilis* showed a mixture of both sources (prob. 30–55% of gut contents).

**Figure 3:**
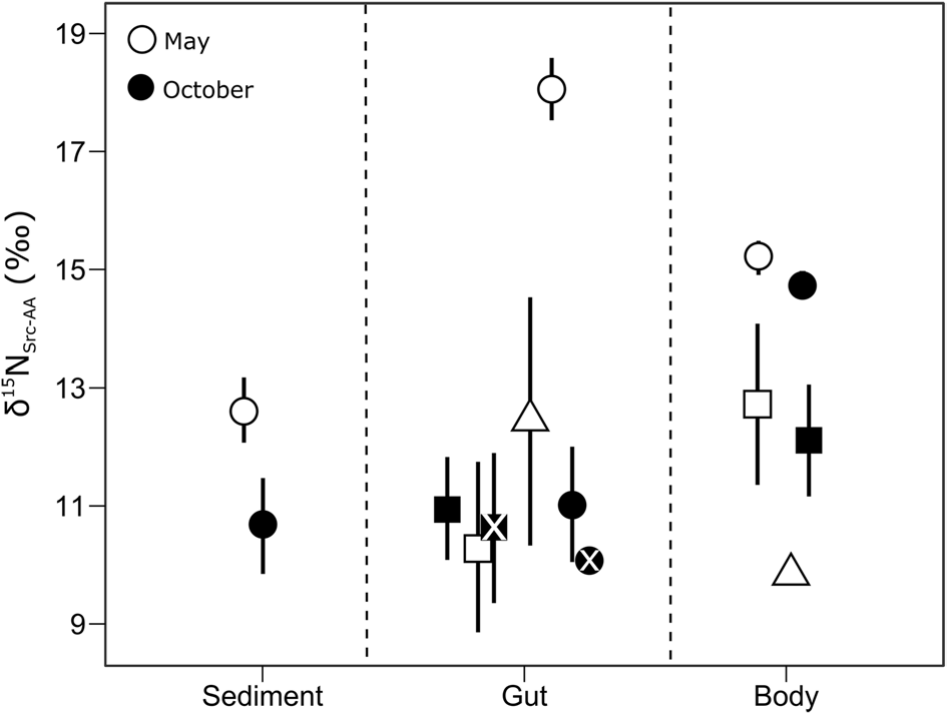
δ^15^N values of “source” AAs (δ^15^N_Src-AA_; mean ± SD) of surface sediments (0–5 mm), gut contents and body tissue collected in the period of low food supply (i.e. May; open symbols) and in the period of high food supply (i.e. October; closed symbols). Gut contents (frontgut: crossed symbol; hindgut: plain symbol) and body tissue correspond to species of deposit feeders: *O.* *mutabilis* (squares), *S.* *globosa* (triangles) and *E.* *rostrata* (circles).

δ^15^N values of “source” AAs (δ^15^N_Src-AA_) in gut contents and body tissue in *O.* *mutabilis* (11.3 ± 1.3‰; mean ± SD) and *S.* *globosa* (11.1 ± 1.9‰) were similar to surrounding sediments (11.6 ± 1.3‰). However, *E.* *rostrata* body tissue (15.0 ± 0.3‰) and gut contents in the period of low food supply (18.1 ± 0.5‰) showed higher δ^15^N_Src-AA_ values relative to sediments (Fig. 3).

### Degree of degradation of sediments and gut contents

DI values were significantly higher (i.e. less degraded) in gut contents of *O.* *mutabilis* (0.34 ± 0.07) and *S.* *globosa* (0.43 ± 0.42) than in those of *E.* *rostrata* (-0.97 ± 0.31; Tukey test, P < 0.01; Table 2). The lower %Mol Gly in gut contents of *O.* *mutabilis* (18.8 ± 1.71%) and *S.* *globosa* (13.3 ± 3.3%) also indicated a lower degree of degradation than in those of *E.* *rostrata* (45.0 ± 7.0%; Tukey test, P < 0.01; Table 2)*.* DI values in sediments were higher in the period of low food supply (− 0.030) than in that of high food supply (− 0.94). Σ*V* values ranged between 2.6 and 4.9, indicating a high degree of heterotrophic microbial resynthesis. However, Σ*V* values were not significantly different among species or between species and sediments (Tukey test, P > 0.05; Table 2).

**Table 2:** Degradation index (DI), Σ*V* parameter, and %Mol Gly in surface sediments (0–5 mm) and gut contents (F: foregut; H: hindgut) of deposit feeders (mean ± SD) collected in periods of low food supply (i.e. May) and high food supply (i.e. October). The sample size was two for each mean value, and for those values not including standard deviation the parameter was calculated from one sample.

		DI		Σ*V*		%Mol Gly	
		Low food supply	High food supply	Low food supply	High food supply	Low food supply	High food supply
Sediment		-0.030 ± 0.026	-0.94 ± 0.18	2.7	3.2 ± 0.3	15.9 ± 10.1	32.9 ± 4.5
*O.* *mutabilis*	F		0.15 ± 0.18		2.7 ± 0.01		19.2 ± 0.6
	H	0.33 ± 0.07	0.42 ± 0.05	4.0 ± 1.3	2.8 ± 0.2	20.5 ± 0.6	16.9 ± 0.8
*S.* *globosa*	F						
	H	0.43 ± 0.42		3.2		13.3 ± 3.6	
*E.* *rostrata*	F		-0.88 ± 0.24		3.2		38.4 ± 0.2
	H	-0.82 ± 0.38	-1.21 ± 0.31		3.0 ± 0.04	49.8 ± 4.5	46.9 ± 9.3

## Discussion

Here we used CSIA-AA to show that megafaunal deposit feeders (DF) were secondary consumers of detritus on the abyssal plain at Station M. This was shown by their trophic position, which was two trophic levels higher than the surrounding sediments. To this date, these are the first estimates of trophic position reported for deep-sea DFs using CSIA-AA approaches. These estimates were higher than expected for organisms feeding mostly on detritus, which are assumed to have trophic positions consistent with primary consumers. Previous research using stable isotope analysis of bulk material has reported high δ^15^N values in DFs relative to sediments, attributing such values to feeding selection^35–38^, to feeding on older, more recycled material^35^ or to a different trophic ^15^N-discrimination of ingested microbial biomass^37^. However, our results based on CSIA-AA, which are not dependent on assumptions of the δ^15^N value at the base of the food web, indicate that they are secondary consumers. In fact, this was true for the 3 species analyzed, despite their bulk δ^15^N values differing by 3.5‰, which typically corresponds to a difference of one trophic level^39^. Abyssal DFs are, therefore, trophically equivalent to zooplanktivorous fish or insectivorous birds.

Consequently, the main food source of DF must have a trophic position consistent with primary consumers. This agrees with the estimated trophic position of gut contents, which was around one trophic level higher than that of surface sediments (Fig. 1). One possible explanation is that DFs are highly selective of food from the environment, so their diet could be based on selected meiofauna, or metazoan remains, like zooplankton carcasses sinking from the water column. There is an extensive body of research addressing selectivity of abyssal benthic megafauna through time-lapse photography^17^, presence of phytopigments like chlorophyll *a* in the guts^20,40–42^, higher concentrations of organic carbon and nitrogen in their guts compared to surrounding sediments^18^, or excess ^234^Th activities in gut contents similar to sediment traps but higher than surface sediments^21,43^. In our study, the gut contents were 18 to 35 times enriched in N from AAs relative to sediments (Table 1), so the guts of DFs concentrate material highly enriched in AAs. However, despite the differences observed in trophic position (estimated from δ^15^N values of specific AAs), δ^15^N and δ^13^C values of bulk gut contents were not different from those of sediments (but see below for differences among species). If the material present in the guts had been selected and ingested from the environment, their high TP relative to sediments would have been reflected in higher bulk δ^15^N values as well^4^. However, ^15^N was not gained nor lost in the gut. This unchanged isotopic signal of bulk material can only be explained if the increase in TP of gut contents took place after the particles had been ingested. In other words, our results suggest that the estimated TP in the guts corresponded mainly to a living microbial biomass, whereas the bulk δ^15^N values remained unchanged because they integrated the living biomass with waste products, which became depleted in ^15^N but do not include AAs.

A remarkable abundance of bacteria in tentacles^44^ and guts^26^ of abyssal holothurians has been previously reported, with abundances 1.5-fold to fivefold higher than the surrounding sediments^19,23,24^. Also, a high RNA:DNA ratio found in the foregut of *Oneirophanta* *mutabilis*^40^ supported the idea of a high proliferation of bacteria in their guts. Although this microbial community could be selected from the environment, our hypothesis that they primarily grow inside the gut was supported by δ^13^C values of essential AAs (δ^13^C_EAA_). We found that surface sediments and gut contents had distinct δ^13^C_EAA_ patterns (Fig. 2), suggesting that they are composed of different microbial communities because only organisms able to synthesize the essential AAs de novo generate distinct δ^13^C_EAA_ fingerprints^45^. In this regard, Amaro et al.^25^ found that bacteria present in the guts of the abyssal holothurian *Molpadia* *musculus* showed a 73% dissimilarity with the surrounding sediments and ca. 40% of bacterial OTUs (Operational Taxonomical Units) were associated uniquely with the guts. Overall, guts of mobile megabenthos concentrate large quantities of organic matter that are largely the living biomass of heterotrophic prokaryotes that form a unique community in the guts.

It is noteworthy that the TP of foregut contents was similar to that of hindguts (Fig. 1). Moreover, despite the decrease in %TN and %TOC along the digestive tract, the contribution of N from AAs remained unchanged (Table 1). This supports the presence of an important microbial community in different sections of the digestive tract. Previous research also reported higher bacteria counts in the foregut and hindgut of deep-sea DFs, with decreased numbers in the midgut^23,24,26^, where most absorption of nutrients occurs^46^. The presence of a flourishing microbial community in hindguts of DFs, later released as fecal pellets to the environment, might create microbial hot spots and contribute to the microbial productivity on the abyssal plain.

The functional role of prokaryotes in the guts of abyssal DFs is largely unknown. It has been suggested that they might be used as a nutritional source since DFs assimilate fatty acids of bacterial origin^47–49^. However, using counts of bacteria in the guts, the calculated prokaryotic production and the gut transit time, it has been estimated that, overall, prokaryotic biomass contributes only between 0.1–3% of the total protein taken up by deep-sea holothurians^24,25,27^. In our study the trophic position estimated for the studied DFs, one trophic level above their gut contents, led us to hypothesize that DFs were assimilating AAs from the prokaryotic biomass inhabiting the guts. However, δ^13^C values of essential AAs in the body tissue of DFs, which come necessarily from the diet, were a combination of those of sediments and gut contents, pointing also to the assimilation of AAs directly from ingested detritus (Fig. 2). It is also possible that the AAs present in the guts could be part of secreted echinoderm digestive enzymes or from the lysis of cells associated with the death of the animals during collection^50^. However, the differences in δ^13^C_EAA_ pattern between gut contents and body tissue confirms that only a minority of gut content AAs derived from the DFs. Our results suggest caution on making the assumption that the biomass of a sea cucumber is a good isotopic proxy for detritus^51^.

We also found differences between the three species analyzed, despite the general patterns described. The elasipod holothurians *O.* *mutabilis* and *S.* *globosa* are two of the fastest mobile megafauna at Station M^52^, and they cover a large surface area of the seafloor [294.2 cm^2^ h^−1^ by *O.* *mutabilis* and 59.8 cm^2^ h^−1^ by *S.* *globosa*^52^]. Both species are surface DFs^53^ that have simple tubular guts which indicate that they feed continuously^23,24^. On the other hand, the echinoid *E.* *rostrata* moves at a lower speed but due to its large size also covers large areas (45.7 cm^2^ h^−1^; ^52^). *Echinocrepis* *rostrata* also carves trails at a depth range of 0–2 cm^54^, so it is likely that they ingest sediment below 0.5 cm. However, this was not reflected in their isotopic composition because sediments showed a similar isotopic composition downcore (Supplementary material, Table S2). *E.* *rostrata*, like most echinoids, have compartmentalized digestive tracts^55^, which allows prolonged residence times and potentially the digestion of more refractory organic matter. Hence, due to its ability to cover larger areas of the seafloor, *O.* *mutabilis* was expected to be the most selective species, which was in agreement with our results of the highest %TN and bulk δ^15^N values in their foreguts compared to the surrounding sediments (Table 1, Fig. 1). In addition, gut contents of *O.* *mutabilis* and *S.* *globosa* were less degraded (i.e. higher DI and lower Mol% of Gly) than gut contents of *E.* *rostrata*, supporting the selection of fresher organic matter by the two holothurians.

The Σ*V* parameter, which is an index for microbial heterotrophic resynthesis^56^, was comparable between gut contents of the three species and sediments (Table 2). The broad array of metabolic pathways for AAs used by heterotrophic microbes generate scattered δ^15^N patterns in “trophic” AAs that can be quantified by increases in the Σ*V* parameter^56^. For that reason, it would be expected that microbial resynthesis within the guts would yield higher Σ*V* values than in sediments. However, the guts might provide a stable environment for a unique microbial community that could be using specific metabolic pathways. Thus, our results suggest that the environment within the guts resembled that of a specific bacterial culture, which led to changes in δ^15^N values of trophic AAs similar to those of metazoans^29,57^.

We further investigated potential seasonal changes in the trophic identity of DFs by collecting samples in two periods with contrasting environmental conditions. At Station M, there is a marked seasonality in the flux of particulate organic carbon reaching the abyssal plain, with a higher density of detrital aggregates arriving from June through December^58^. A higher cover of phytodetritus was also observed on the seafloor in October than in May the year we sampled (Smith, unpublished data). The seasonality of food supply has an effect on the abundance^14,59^ and reproduction^54^ of megafauna, but the overall trophic identity of the studied DFs as secondary consumers was maintained. However, we observed some differences in the isotopic composition of gut contents. Gut contents of both *O.* *mutabilis* and *E.* *rostrata* showed higher bulk δ^15^N values during the period of low food supply (i.e. May). For *O.* *mutabilis* that difference appeared to be trophic (change in “trophic” AAs but not “source” AAs; Fig. 1), probably due to the consistently larger size of specimens collected in May relative to those collected in October (Supplementary material, Table S1). Larger individuals can be more selective by covering larger areas, and their longer gut might allow a more abundant microbial community to grow within it. This difference in TP of gut contents was also reflected in *O.* *mutabilis* body tissue, which was slightly higher in the period of low food supply and higher than in the other two species. On the contrary, for *E.* *rostrata* the seasonal difference in bulk δ^15^N values of gut contents was associated with changes in “source” AAs, since the δ^15^N_Src-AA_ values of gut contents collected in the period of low food supply were higher than those of sediments (Fig. 3). Increases in δ^15^N_Src-AA_ values are usually identified with extracellular enzymatic hydrolysis of proteins from detrital pools by microbes^34,60^ because ^14^N-containing bonds are preferentially cleaved, leaving higher δ^15^N values of AAs in the detrital pool. This suggests that during the period of lower food supply urchins were more dependent on the microbial community within the guts that hydrolyzed proteins from highly degraded detritus.

Despite the higher cover of phytodetritus observed on the seafloor in October than in May (Smith, unpublished data), such differences were not reflected in the elemental or stable isotopic composition of surface sediments. In fact, the DI, the Σ*V* parameter and the %Mol Gly indicated that sediments collected in the period of high food supply (i.e. October) were slightly more degraded (Table 2). It is likely that our sediment samples, randomly collected from the seafloor, did not comprise patches of freshly deposited aggregates during the more productive period. Nevertheless, the seasonal differences were reflected in the assimilation of essential AAs by DFs. During the period of low food supply (i.e. May) the body tissue of all three species had δ^13^C_EAA_ values that resembled those of gut contents, whereas during the more productive period (i.e. October) they were more similar to those of sediments in *E.* *rostrata* and a mixture of both in *O.* *mutabilis* (Fig. 2B)*.* Previous findings showed a shift in the diet of *O.* *mutabilis* from fresh to more refractory material in response to seasonal variations in food availability^20,61^. Our results suggest that when there is a higher abundance of detritus available, the essential AAs can be obtained from the environment, but in periods of food scarcity the microbial community within the guts might be important for the sustenance of DFs. This is in line with findings suggesting that the development of a specific gut flora in a deep-sea holothurian is enhanced by a low organic content in sediments^62^. Moreover, this was true for *E.* *rostrata* and *O.* *mutabilis,* which are two species with very different feeding modes, implying that the importance of the microbial community present in the guts of abyssal DFs might be widespread. Despite the consistency in our data, we caution that our results are based on a low number of samples per season and more studies are needed to fully understand how nutritional sources for abyssal megafauna vary due to seasonal differences in the food supply.

Our study revealed for the first time that DFs inhabiting the abyssal plain are secondary consumers of detritus, in contrast to the common assumption that their nutrition comes directly from detritus. The gut contents of the three species analyzed are trophically distinct from the surrounding surface sediment and their mix of prokaryotic biomass and detritus form a unique food source, which is one trophic level higher than sediments. Our results suggest that gut contents are mainly composed of a heterotrophic microbial community, which is assimilated as a food source by the DFs most importantly in periods of low food supply. In summary, we found that microbial communities in the guts of deposit feeders play a key role as primary consumers in the food web of the abyssal plain. The trophic dynamics in this deep-sea ecosystem are bottom-up controlled by the flux of organic matter^9^. In this ecological framework, the role of prokaryotes as primary consumers might be crucial in providing stability to the food web. As a result, gut microbes deserve attention in future studies analyzing energy flows in the food web of abyssal plains.

## Methods

### Sample collection

Samples were collected from the abyssal plain (~ 4000 m) in the Eastern North Pacific off of the California coast (34.50° N, 123.06° W; Station M). Sediment cores and megafauna were collected in May and October 2019 using the HOV *Alvin* and the ROV *Doc* *Ricketts*, respectively. Upon retrieval to the surface, samples were placed in a cool room (5 °C) for their further processing.

Sediment cores of 7 cm diameter were sliced in depth intervals (0–0.5, 0.5–1, 1–2, 2–3, 3–4, 4–5 and 5–10 cm), placed in petri dishes and stored frozen at − 80 °C. Specimens of holothurians *Oneirophanta* *mutabilis* complex, *Scotoplanes* *globosa* and echinoid *Echinocrepis* *rostrata* were weighed and measured, then they were dissected using an scalpel. We chose for this study three of the most abundant species of deposit feeders at Station M ^14,17,54,59^ with a large size so that we could sample the amount of gut contents required for the analyses (~ 150 mg in dry weight). We made a longitudinal cut along the digestive tract and took a sample of the foregut and hindgut contents, avoiding gut tissue. Then we removed the remaining guts and took a sample from cleaned body tissue, or in the case of echinoids, from the test. All samples were placed in cryovials and frozen in liquid nitrogen, and subsequently stored at − 80 °C.

### Stable isotope analyses

Samples of sediments, gut contents and body tissue were freeze dried and ground to a homogenous powder using mortar and pestle. For analysis of bulk nitrogen and carbon isotopic composition, TN and TOC, ~ 0.7 and 3 mg of body tissue from holothurians and echinoids, respectively, ~ 5 mg of gut content and ~ 20 mg of sediments were placed in silver capsules. Samples were acidified to remove carbonates with 1 M HCl, which was added dropwise until bubbling ceased, then dried at 60 ºC and packed. Nitrogen and carbon elemental composition and isotopic composition were determined simultaneously using an isotope ratio mass spectrometer (DeltaPlusXP or Delta-V-Advantage) coupled to an elemental analyzer (Costech Model 4010). Measurement error based on within-run replicates of reference materials (glycine and homogenized fish tissue, both extensively characterized with NIST-certified reference materials and their δ^13^C and δ^15^N values verified independently in other laboratories) were typically of the order of less than ± 0.2‰ for δ^15^N and δ^13^C values. In elemental analyses, the variation of repeated measures performed in a subset of samples was < 10%.

For δ^15^N and δ^13^C analysis of individual AAs, we analyzed a subset of two replicates for each type of sample and each species from both cruises, except for *S.* *globosa*, which was not found in October (sediments: n = 4, gut content: n = 14, body tissue: n = 10). Samples were analyzed following the methods of Hannides et al.^60^. Briefly, samples were hydrolyzed using trace metal-grade 6 N HCl and then purified using cation exchange chromatography. Samples were then esterified using 4:1 isopropanol:acetyl chloride and derivatized using 3:1 methylene chloride:trifluoroacetyl anhydride. To account for carbon added during derivatization and variability of isotope fractionation during analysis, we also derivatized and analyzed a sample containing a set of 14 pure AAs (all amino acids analyzed in the samples, see list below) purchased commercially (Sigma-Aldrich, St. Louis, Missouri, USA). The resulting trifluoroacetyl and isopropyl ester derivatives were purified using chloroform extraction and stored at − 20 °C until analysis.

Carbon isotope composition was measured using a Thermo-Fisher Scientific MAT 253 isotope ratio mass spectrometer interfaced with a Trace Ultra GC-III via ConFlo IV. δ^13^C values were corrected based on the analysis of the set of pure AAs prepared under the same conditions following the approach by Silfer et al.^63^. Nitrogen isotopic composition of AAs was analyzed using a Thermo Scientific Delta V Plus IRMS interfaced to a trace gas chromatograph (GC) fitted with a 60 m BPx5 capillary column through a GC-C III combustion furnace (980 °C), reduction furnace (680 °C) and liquid nitrogen cold trap.

When possible, each sample was measured on 3 replicate injections (but sediments were only injected once due to their low AA content), with internal reference materials norleucine and aminoadipic acid with known isotopic composition co-injected on each run. The accuracy of CSIA-AA measurements, as the difference between the isotopic composition of the internal reference materials co-injected on each run and their known δ^15^N and δ^13^C values, was typically < 1‰. For replicate injections, δ^15^N and δ^13^C standard deviations averaged 0.4‰ and 0.5‰, respectively. Results for each sample are given as the mean values of all injections. We obtained information for the following AAs: alanine (Ala), aspartic acid (Asx; included the contribution of asparagine), glycine (Gly), glutamic acid (Glx; included the contribution of glutamine), isoleucine (Ile), leucine (Leu), lysine (Lys), methionine (Met), phenylalanine (Phe), proline (Pro), serine (Ser), threonine (Thr), tyrosine (Tyr), and valine (Val). Full AA reference suites of known weight were injected every 3 sample injections. The corresponding response factors (Vs [nmol AA]^− 1^) were used to determine total AA concentration and the contribution of each AA to the total AA pool (i.e. Mol% AA; mol AA*i*/Σ mol AA × 100% for each AA *i*). δ^15^N values were normalized using the fitted regression line between the known δ^15^N_AA_ values and the measured δ^15^N_AA_ values of the AA reference suite injected prior and after every 3 sample injections.

### Stable isotopes data analysis

δ^15^N values of “source” AAs (δ^15^N_Src-AA_) were calculated as the average δ^15^N value of: serine, phenylalanine, lysine and glycine. Trophic position (TP) was calculated using the equation from Chikaraishi et al.^31^: TP = (δ^15^N_glx_ – δ^15^N_phe_ – 3.4)/7.6 + 1, where δ^15^N_glx_ and δ^15^N_phe_ are the δ^15^N values of glutamic acid and phenylalanine, respectively, 3.4 ± 1.0‰ is the difference between glutamic acid and phenylalanine in primary producers (β), and 7.6 ± 1.2‰ is the trophic discrimination factor (TDF_AA_). Uncertainty in the calculation of δ^15^N_Src-AA_ and TP due to analytical error and uncertainties in TDF and β values was calculated by propagation of errors (^64^; Supplementary material, Table S1). To determine whether sediments and gut contents constituted distinct food sources we focused on δ^13^C values of essential AAs (Val, Thr, Ile, Leu, Phe and Lys). We normalized the δ^13^C values of each essential AA to the mean value of all essential AAs for each sample. Algae and bacteria have highly conserved modes of amino acid biosynthesis that produce unique patterns of carbon isotope fractionation. This allows the origins of essential amino acids to be determined from a comparison of the distribution patterns of δ^13^C values between samples since they fall on the same scale around an average equal to zero^45^.

The Σ*V* parameter is a proxy for heterotrophic resynthesis of AAs and is defined as the average deviation of δ^15^N values of individual trophic AAs from the mean δ^15^N value of those AAs^56^. The Σ*V* was calculated as: Σ*V* = 1/*n* Σ Abs (χ_*i*_), where *n* is the total number of AAs used for the calculation and χ_*i*_ is the deviation of the δ^15^N value of amino acid *i* from the mean δ^15^N value of the *n* amino acids [ δ^15^N_*i*_ – (Σ δ^15^N_*i*_ /*n*)]. We used five AAs to calculate Σ*V* (Ala, Leu, Pro, Asx and Glx), excluding Ile because its δ^15^N value was missing in a few samples. Nevertheless, for those samples with estimated δ^15^N values of the six AAs the Σ*V* parameter was not significantly different when estimated with and without Ile since the slope of the Σ*V*_*6-AA*_ *vs.* Σ*V*_*5-AA*_ relationship (n = 18, R = 0.96, P < 0.001) was not significantly different from 1 [slope = 0.98 (95% confidence interval 0.87, 1.09)].

The degradation index (DI) is based on the selective preservation of AAs, so that AAs that comprise refractory material like cell walls will be more abundant in degraded material^56,65^*.* In this sense, Gly is abundant in cell walls so its high molar abundance (Mol% Gly) also indicates higher degradation state of particles or sediments^65,66^. The DI was calculated following the formula proposed by Dauwe et al.^65^: DI = Σ [(var_*i*_ – AVG var_*i*_ )/STD var_*i*_] × fac.coef_*i*_, where var_*i*_ is the Mol% of amino acid *i* in our dataset*,* AVG var_*i*_ and STD var_*i*_ are its mean and standard deviation in the reference dataset (based on a variety of samples from marine phytoplankton to deep-sea sediments) and fac.coef_*i*_ the factor coefficient from amino acid *i* based on the first axis of the PCA. AVG var_*i*__*,*_ STD var_*i*_ and fac.coef_*i*_ were obtained from Table 1 in Dauwe et al.^65^.

### Data analysis

We used analysis of variance (ANOVA) to study differences in TP increment or bulk δ^15^N values between types of samples (i.e. sediments, foregut, hindgut, and body tissue). To disentangle the differences in patterns of δ^13^C values of EAAs (Val, Thr, Ile, Leu, Phe and Lys) we used multivariate statistics. We performed a principal component analysis (PCA) to the normalized δ^13^C values of each AA, then we used the permutational analysis of variance (PERMANOVA) to find statistically significant differences in δ^13^C values of EAAs between groups. We also performed a Linear Discriminant Analysis (LDA) using δ^13^C_EAA_ values of sediments and gut contents as a training set to determine whether δ^13^C_EAA_ values of body tissue grouped more closely with sediments or gut contents. All analyses were performed using R version 3.6.3^67^.

## Supplementary Information

Supplementary Figures.

## Data Availability

Data for individual AAs are available at BCO-DMO: https://www.bco-dmo.org/dataset/840749.
