# Supplementary material for: Abyssal deposit feeders are secondary consumers of detritus and rely on nutrition derived from microbial communities in their guts
Source: Sci Rep. 2021 Jun 15;11:12594. doi: 10.1038/s41598-021-91927-4 (PMC8206261; doi:10.1038/s41598-021-91927-4)
Supplement: Supplementary file 1 — Supplementary Figures. [file 41598_2021_91927_MOESM1_ESM.pdf]

Deposit feeders are secondary consumers of detritus in an abyssal food web

Sonia Romero-Romero, Lee C. Miller, Jesse Black, Brian N. Popp, Jeffrey C. Drazen

Supplementary Information

Table S1. Stable isotopic composition: δ15N, δ13C, and δ15NSrc-AA values; elemental composition: total nitrogen (TN), contribution of total nitrogen from amino acids (N in AA); trophic position (TP); and degree of degradation: ΣV, DI, Mol% of Gly of individual samples used in the study. Mean ± SD and values in parenthesis represent the propagated error for δ15NSrc-AA values and trophic position (TP) calculation.

Sample ID	Type	Species	Month	Weight (g)	$\delta^{15}N$ (‰)	$\delta^{13}C$ (‰)	TN (wt. %)	N in AA (wt. %)	$\delta^{15}N_{Src-AA}$ (‰)	TP	Σ*V*	DI	Mol Gly (%)
AT4210_4_0-0.5	Sediment		May		9.7	-22.1	0.23	$8.26\times10^{-4}\pm8.71\times10^{-4}$	13			-0.048	8.8
AT4210_2_0-0.5	Sediment		May		9.7	-22.1	0.22	$5.34\times10^{-4}\pm5.98\times10^{-4}$	12.2	0.9	2.7	-0.011	23.1
Pulse72_57_0-0.5	Sediment		Oc		8.8	-22.1	0.24	$3.82\times10^{-4}\pm3.69\times10^{-4}$	11.2	0.8	3.0	-1.068	36.1
Pulse72_54_0-0.5	Sediment		Oc		8.5	-22.2	0.24	$9.62\times10^{-4}\pm8.49\times10^{-4}$	10.1	1.4	3.4	-0.81	29.7
AT4210_71	Hindgut	*E. rostrata*	May		11.3	-21.1	0.46	8.10×10^{-3}	18.5 (0.8)	2 (0.3)		-0.55	46.6
AT4210_75	Hindgut	*E. rostrata*	May		9.7	-22.0	0.32	$7.35\times10^{-3}\pm4.72\times10^{-3}$	17.7	1.2		-1.085	53
Pulse72_8	Foregut	*E. rostrata*	Oc		8.2	-21.2	0.54	3.70×10^{-2}				-0.72	38.5
Pulse72_12	Foregut	*E. rostrata*	Oc		7.50	-21.5	0.35	$4.14\times10^{-2}\pm6.68\times10^{-3}$	10.1	1.8	3.2	-1.05	38.2
Pulse72_10	Hindgut	*E. rostrata*	Oc		7.10	-21.8	0.37	$1.80\times10^{-2}\pm3.06\times10^{-3}$	11.7	1.9	2.9	-0.99	40.4
Pulse72_14	Hindgut	*E. rostrata*	Oc		8.00	-21.6	0.34	$5.89\times10^{-2}\pm3.26\times10^{-3}$	10.3 (2.2)	2.1 (0.3)	3.0	-1.43	53.5
AT4210_109	Hindgut	*S. globosa*	May		9.6	-23.0	0.29	4.18×10^{-3}	13.9 (1.2)	1 (0.2)		0.72	10.7
AT4210_105	Hindgut	*S. globosa*	May		10.6	-22.1	0.34	2.15×10^{-2}	11 (0.8)	2.3 (0.4)	3.2	0.13	15.8
AT4210_14	Hindgut	*O. mutabilis*	May		9.1	-23.6	0.28	$3.75\times10^{-2}\pm2.34\times10^{-2}$	11.3 (1.4)	1.9 (0.3)	3.1	0.28	20

AT4210_18	Hindgut	*O. mutabilis*	May		12.5	-21.9	0.66	$5.57\times10^{-2}\pm4.00\times10^{-3}$	9.3 (0.9)	3.0 (0.5)	4.9	0.38	20.9
Pulse72_5	Hindgut	*O. mutabilis*	Oc		7.9	-21.8	0.36	$2.13\times10^{-2}\pm2.65\times10^{-3}$	11.6 (0.6)	2.1 (0.3)	2.9	0.46	17.4
Pulse72_46	Hindgut	*O. mutabilis*	Oc		8.2	-21.3	0.55	2.82×10^{-2}	10.3 (0.7)	2.3 (0.4)	2.7	0.39	16.3
Pulse72_3	Foregut	*O. mutabilis*	Oc		11.8	-21.7	0.91	$6.55\times10^{-2}\pm1.17\times10^{-3}$	11.5 (0.7)	2.5 (0.4)	2.7	0.28	18.8
Pulse72_44	Foregut	*O. mutabilis*	Oc		9.9	-21.9	0.75	$3.22\times10^{-2}\pm7.01\times10^{-3}$	9.7 (0.7)	1.9 (0.3)	2.7	0.011	19.6
AT4210_68	Body	*E. rostrata*	May	145.6	16.1	-20.8	1.67	0.38±0.11	15.4 (1.9)	2.6 (0.5)	3.1	-1.27	54
AT4210_72	Body	*E. rostrata*	May	138.1	15.1	-18.7	2.13	0.32±0.1	15 (1)	3.0 (0.5)	3.3	-1.33	45.5
Pulse72_7	Body	*E. rostrata*	Oc	229.5	14.6	-18.7	0.90	0.15±0.03	14.9 (0.9)	3.1 (0.5)	2.8	-1.66	63.3
Pulse72_11	Body	*E. rostrata*	Oc	127.5	14.9	-18.9	0.95	0.15±0.05	14.6 (1.1)	3.0 (0.5)	3.0	-1.42	57.5
AT4210_102	Body	*S. globosa*	May	39.0	13.5	-18.7	3.43	1.36±0.24	9.9 (0.6)	2.5 (0.4)	2.3	-0.40	27.3
AT4210_106	Body	*S. globosa*	May	37.5	13.5	-18.2	3.47	0.92±0.29	9.7 (0.5)	2.6 (0.4)	2.3	-0.45	28.3
AT4210_11	Body	*O. mutabilis*	May	119.3	16.9	-20.2	3.73	0.96±0.20	11.7 (0.8)	3.3 (0.5)	2.0	-0.73	36.6
AT4210_15	Body	*O. mutabilis*	May	67.7	16.8	-20.3	3.14	0.65±0.21	13.7 (0.4)	3.3 (0.5)	2.5	-0.62	33.8
Pulse72_2	Body	*O. mutabilis*	Oc	46.1	14.6	-19.2	3.42	1.28±0.42	12.8 (1.1)	2.5 (0.4)	1.3	-0.79	35.5
Pulse72_43	Body	*O. mutabilis*	Oc	31.1	15.2	-19.8	3.23	1.33±0.53	11.4 (1.3)	3.1 (0.5)	2.5	-0.82	35.7

Table S2. Stable isotopic composition and elemental composition of sediment samples downcore.

Month	Depth (mm)	$\delta^{15}N$ (‰)	$\delta^{13}C$ (‰)	TOC (wt. %)	TN (wt. %)
May	0-5	9.7±0.0	-22.1±0.1	1.37±0.05	0.22±0.01
	5-10	9.6±0.1	-22.1±0.1	1.42±0.05	0.23±0.01

	10-20	9.6±0.1	-22.1±0.0	1.46±0.07	0.24±0.01
	20-30	9.5±0.0	-22.1±0.0	1.51±0.08	0.24±0.01
	30-40	9.4±0.1	-22.1±0.0	1.57±0.02	0.25±0.00
	40-50	9.4±0.1	-22.2±0.1	1.55±0.06	0.25±0.01
	50-100	9.5±0.1	-22.2±0.2	1.53±0.03	0.24±0.00
October	0-5	8.8±0.3	-22.2±0.1	1.55±0.10	0.23±0.01
	5-10	8.6±0.3	-22.2±0.1	1.48±0.05	0.19±0.00
